# Complications in Distal Minimally Invasive Metatarsal Osteotomies: Systematic Review and Meta-Analysis

**DOI:** 10.3390/medicina61081435

**Published:** 2025-08-09

**Authors:** Angélica María Fernández-Gómez, Eduardo Nieto-García, Leonor Ramírez-Andrés, Juan Vicente-Mampel, Javier Ferrer-Torregrosa

**Affiliations:** 1 Podiatry Department, Faculty of Medicine and Health Sciences, Valencia Catholic University San Vicente Mártir, 46001 Valencia, Spain; angelicamaria.fg@mail.ucv.es (A.M.F.-G.); eduardo.nieto@ucv.es (E.N.-G.); leonor.ramirez@ucv.es (L.R.-A.); javier.ferrer@ucv.es (J.F.-T.); 2 Department of Physiotherapy, School of Medicine and Health Science, Catholic University of Valencia, 46001 Valencia, Spain

**Keywords:** complications, DMMO (distal minimally invasive metatarsal osteotomy), metatarsalgia, meta-analysis, MIS (minimally invasive surgery)

## Abstract

*Background and Objectives*: Minimally invasive distal metatarsal osteotomy (DMMO) has established itself as an effective surgical technique for the treatment of metatarsalgia, notable for its reduced postoperative pain and faster recovery. However, doubts remain regarding the frequency and nature of postoperative complications. The objective of this systematic review and meta-analysis was to evaluate the incidence of the most frequent complications associated with the DMMO technique, including prolonged edema, delayed bone healing, transfer metatarsalgia, floating toe, and persistent pain. *Materials and Methods*: A systematic review was conducted following the PRISMA 2020 guidelines, with the protocol registered in the PROSPERO database (CRD420251067666). Searches were conducted in the PubMed, Scopus, and Web of Science databases, including clinical studies published between 2010 and 2025. The inclusion criteria covered studies in adults treated with DMMO and reporting postoperative complications. The methodological quality assessment was performed using Joanna Briggs Institute (JBI) tools according to the design of each study. Random-effects models were used for the meta-analyses, assessing heterogeneity using the I^2^ statistic. *Results*: Fifteen studies with a total sample of more than 493 patients were included. Prolonged edema was the most common complication (30.91%), followed by delayed bone healing (14.9%), transfer metatarsalgia (12.73%), floating toe (10.45%), and persistent pain (8.5%). Less frequent complications included nonunion, infections, necrosis, and bone misalignments. The combined incidence of floating toe was 40% (I^2^ = 0%), while prolonged edema showed considerable heterogeneity (I^2^ = 88.3%). The overall quality of the evidence was considered moderate to low, mainly due to the predominance of observational studies. *Conclusions*: The DMMO technique represents a minimally invasive surgical option with generally favorable results. However, some complications, such as prolonged edema and floating toe, have a significant incidence. The methodological variability between studies highlights the need for standardized protocols and higher-quality prospective studies to establish the safety profile of this technique more accurately.

## 1. Introduction

Metatarsalgia is one of the most common conditions affecting the foot, causing pain mainly under the heads of the metatarsals [1]. This condition is especially prevalent in individuals with biomechanical abnormalities in the foot [2,3]. The causes of metatarsalgia can be diverse and multifactorial [4]. Biomechanical alterations are the main etiological factor [5]. The prevalence of this condition is estimated at approximately 10% of the population [1], with a higher incidence in women [6], and it is estimated that up to 80% of people may experience symptoms of metatarsalgia at some point in their lives [7,8,9].

Conservative treatment is often effective in many cases, such as foot orthotics [2,10]. However, when symptoms persist or anatomical deformities are severe, surgery becomes the best option to restore normal foot function and relieve pain [2,11]. In this context, metatarsal osteotomies have established themselves as one of the most effective surgical techniques [12,13,14]. Among these, Distal Minimally Invasive Metatarsal Osteotomy (DMMO) has gained great popularity due to its less invasive approach, which means less postoperative pain, faster recovery times, and a lower incidence of serious complications [15,16].

The DMMO technique consists of performing a complete extracapsular osteotomy in the distal metaphyseal area of the metatarsal [12,13,14,17], without the need for fixation materials such as screws or plates. It is carried out through a small incision using fluoroscopic guidance and controlled burring, allowing for shortening and/or dorsiflexion of the bone to redistribute plantar pressure. This procedure aims to improve alignment and load distribution across the forefoot. The difference from other surgical techniques is that they are performed through small incisions [18] and are supported by fluoroscopic imaging of the bone structures [19], allowing for greater safety, precision, and less risk of affecting soft tissues. As it is a minimally invasive procedure, patients have a faster recovery and, generally, a quicker return to normal life.

DMMO osteotomy is a valuable tool, but it is essential that professionals have a thorough understanding of the most common risks and complications in order to act appropriately when they arise. In this paper, we will analyze the complications associated with DMMO, providing a critical overview of the preventive and therapeutic strategies that can help mitigate these risks.

Postoperative complications associated with the Minimally Invasive Distal Metatarsal Osteotomy (DMMO) technique are a key aspect evaluated in this study. Although DMMO is a minimally invasive technique that seeks to reduce complications and speed recovery compared to open procedures [18], it is not without risks [20,21].

This systematic review was designed with the aim of identifying postoperative complications associated with the DMMO technique, characterizing and classifying the included studies, the frequency of reported complications, and the most common complications according to the reviewed literature.

## 2. Materials and Methods

### 2.1. Study Design

#### Registry of Systematic Review Protocol

To conduct this study, we followed the PRISMA 2020 guidelines for conducting systematic reviews [22]. In addition, we registered the protocol in the PROSPERO database (ID: CRD420251067666).

### 2.2. Eligibility Criteria

#### 2.2.1. Inclusion Criteria

The inclusion criteria required that the selected articles be published between 2010 and 2025 and focus on adult patients over the age of 18 years old who had been treated using the surgical technique known as DMMO. In addition, the studies had to consist of clinical research conducted in humans, including clinical trials, prospective or retrospective studies, or case series with more than one patient. A fundamental requirement was that they provide detailed information on both short- and long-term postoperative complications.

#### 2.2.2. Exclusion Criteria

On the other hand, studies that were single case reports without a structured analysis or clinical follow-up, those that described surgical techniques other than DMMO (unless they offered a direct comparison with it), and those that dealt with minimally invasive osteotomies of the first radius, specifically in cases of hallux valgus (HAV), were excluded.

### 2.3. Search Strategy

The bibliographic search process was carried out in June 2025. The search was conducted using three databases: PubMed, Scopus, and Web of Science (WoS). To ensure a solid and structured search strategy, we used a PICO (Population, Intervention, Comparison, and Outcome) framework, which allowed us to precisely define the inclusion and exclusion criteria for the studies (see Table 1).

#### PICO Question

What are the postoperative complications in patients undergoing minimally invasive forefoot surgery using distal percutaneous osteotomies (DMMO)?

With this process, we created the complete search equation for PubMed and then translated the search strings using the “polyglot search” tool from “sr-accelerator” [23] for each database used (Appendix A).

### 2.4. Data Extraction and Summary

Two evaluators (AFG and E.N-G) independently reviewed the full texts of the articles that had passed the initial selection phase based on title and abstract, applying the established inclusion criteria. A third researcher (JVM) was responsible for conducting a complementary evaluation of the identified studies, refining the inclusion criteria to more precisely define the focus of the review. Differences that arose during the selection process were discussed among the reviewers; in cases where consensus was not reached, a fourth reviewer (JFT) was consulted to make a final decision. The online tool SR-Accelerator Deduplicator (https://www.sr-accelerator.com/#/deduplicator (accessed on 10 June 2025)) was used for the initial detection of duplicates, followed by a manual review using the bibliographic management software Mendeley.

All the information extracted was organized in an Excel spreadsheet (see Table 2), which facilitated statistical and comparative analyses.

### 2.5. Study Coding and Summary

Data extraction was performed by the author of this study using a previously designed spreadsheet. This template included the following main variables: Author/Year, Sample (n), Mean age (range), Average follow-up (range), Intervention, Results Evaluated, Complications reported.

### 2.6. Methodological Quality and Risk of Bias

The risk of bias and methodological quality of the included studies were assessed using specific tools from the Joanna Briggs Institute (JBI) [34], selected according to the design of each study. For prospective cohort studies, a list of 11 items was applied to analyze aspects such as the validity of exposure and outcome measurement, comparability between groups, management of confounding factors, and the quality of follow-up and analysis. In the case of case series [35], a list of 10 criteria was used, focusing on the clarity of the inclusion criteria, the description of the participants, consistency in measurements, and adequate presentation of the results. For the single case study, an 8-item tool was used to evaluate the clinical presentation, diagnostic process, treatment applied, and patient outcome.

In addition, the certainty of the evidence obtained from the meta-analysis was assessed using the Grading of Recommendations Assessment, Development and Evaluation (GRADE) methodology [36], which classifies confidence in the results as high, moderate, low, or very low, based on five domains: risk of bias, inconsistency between studies, indirect evidence, imprecision, and publication bias. Given that most of the studies were observational (cohorts and case series), the quality of the evidence was initially considered low. However, this rating could be adjusted upwards or downwards depending on the presence of factors that strengthened or weakened the results, such as a large observed effect, a clear dose–response relationship, or the absence of possible confounding factors. This assessment allowed the reliability of the findings to be contextualized and the clinical conclusions of the study to be substantiated.

### 2.7. Statistical Analysis

A meta-analysis of proportions was performed to estimate the frequency of postoperative complications associated with the DMMO technique. Given that the included studies presented clinical and methodological variability, a random-effects model was used, which allows for real differences between studies to be considered and offers a more conservative estimate of the combined effect. Heterogeneity was assessed using the I^2^ statistic, with values above 50% interpreted as indicative of substantial heterogeneity. The *p*-value of the heterogeneity test was also reported. The results were represented using forest plots, which show the individual proportions and the combined effect, and funnel plots, used to explore possible publication bias.

All statistical analysis and graphing were performed using JASP software (v.0.19.3, Amsterdam, The Netherlands).

## 3. Results

**Figure 1:**
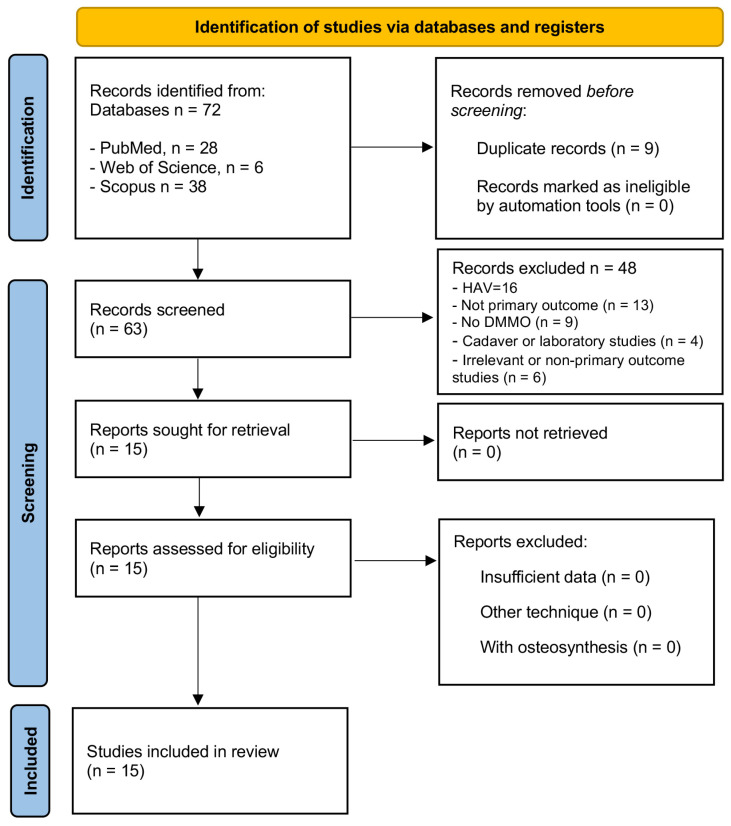
PRISMA flowchart of the article selection process.

In the PRISMA diagram (see Figure 1), we find the different search phases and the results obtained in each of these phases.

A total of 72 articles were initially identified after searching three scientific databases: 28 in PubMed, 38 in Scopus, and 6 in Web of Science. In the first phase, nine duplicate records that appeared in more than one database were eliminated, reducing the total to 61 unique studies for the screening phase.

During the reading of the title and abstract, 48 studies were discarded for not meeting the inclusion criteria focused on the DMMO surgical technique applied to the lesser metatarsals in humans. Among these exclusions, 16 articles addressed pathologies of the first ray, specifically HAV. All of these were excluded because they did not focus on the central rays (second to fourth metatarsals).

Likewise, 13 studies were eliminated because they were not primary research, including systematic reviews, book chapters, or methodological articles without their own clinical data. Nine articles that did not include the DMMO technique as the main intervention were also discarded, as were four studies conducted on cadaveric or laboratory models, which, although they provide anatomical knowledge, do not constitute applicable clinical evidence. Finally, six additional studies were excluded because they presented insufficient or irrelevant data for the objectives of this review (minimal clinical samples or absence of postoperative results).

After this process, 15 articles were selected for full-text reading, which met the established criteria and were included in the systematic review. This process allowed us to form a specific body of evidence, focused on the clinical application of DMMO in the lesser metatarsals, ensuring the relevance, applicability, and quality of the included studies.

### 3.1. Reported Complications

The complications associated with DMMO are highly varied, depending largely on the surgeon’s experience, the clinical indication, and the patient’s profile.

One of the most common complications was postoperative edema, with rates varying depending on the study. Biz et al. (2018) [20] identified transient edema in 29% of cases (27 cases), while Henry et al. (2011) [16] found it in 59% of patients, which is the highest rate reported. Yeo et al. (2016) [18] also described two cases of prolonged edema (15.4%), which disappeared spontaneously, as did Wong et al. (2013) [33], whose adverse events were only edema. Malhotra et al. (2019) [29] and Haque et al. (2016) [25] also noted the presence of edema after 3 months, although it was also transient.

Delayed consolidation was another frequent complication. Biz et al. (2018) [20] observed it in 24.7% of osteotomies (23 cases), although all cases consolidated before 6 months. Magnan et al. (2018) [28] reported two cases in their series of 57 patients, and Salinas-Gilabert et al. (2023) [26] described three cases. Henry et al. (2011) [16] reported a lower incidence, with 5% of cases. De Prado-Ripoll et al. (2021) [21] also documented a single case of delayed consolidation in their sample of 29 patients. Neunteufel et al. (2022) [32] found this complication in 12.9% of cases, and McMurrich et al. (2020) [30] described a delayed union that progressed favorably. In most studies, the delay did not involve complete failure of consolidation or require further intervention.

Regarding transfer metatarsalgia, Biz et al. (2018) [20] documented three cases (3.2%), which required new osteotomies for resolution. Henry et al. (2011) [16] reported 11 patients (29%), and Haque et al. (2016) [25] reported a single case. Malhotra et al. (2019) [29] reported a couple of cases (5%) within their overall complication rate (26%), and Magnan et al. (2018) [28] recorded two episodes. De Prado-Ripoll et al. (2021) [21] reported one mild case that did not require additional treatment. Neunteufel et al. (2022) [32] identified two cases, and Salinas-Gilabert et al. (2023) [26] added another. In contrast, deMeireles et al. (2025) [24] and Wong et al. (2013) [33] did not report transfer metatarsalgia in their respective series.

Transient paresthesias were described exclusively by Biz et al. (2018) [20], with an incidence of 6.4% (6 cases), resolving without intervention. Superficial burns were also observed in the study by Biz et al. (2018) [20] (3.2%) and in that of Magnan et al. (2018) [28], as a result of the use of surgical instruments, especially during the initial learning phase.

With regard to persistent joint stiffness, Biz et al. (2018) [20] identified it in approximately 10% of patients as a mild functional sequela, although these complications were not directly associated with the DMMO technique. In contrast, the study by Yeo et al. (2016) [18] highlighted good preservation of joint range compared to other techniques such as Weil.

More structural complications such as nonunion and malunion were also reported. Haque et al. (2016) [25] described an asymptomatic case of nonunion and another of malunion with persistent metatarsalgia. Malhotra et al. (2019) [29] added an asymptomatic nonunion to their sample. In the study by Krenn et al. (2018) [27], conducted during the learning curve, pseudoarthrosis appeared in 29.6% of cases, reflecting a significant impact of technical mastery on the complication rate.

Bone necrosis was reported in 14.8% of cases by Krenn et al. (2018) [27], also during the early stages of surgical learning. In the rest of the studies reviewed, including those by Henry et al. (2011) [16], Malhotra et al. (2019) [29], and Mehlhorn et al. (2020) [31].

Floating toe was particularly striking in the studies by Neunteufel et al. (2022) [32], with an incidence of 41.9%, and Krenn et al. (2018) [27], with 37%, being mostly asymptomatic. Mehlhorn et al. (2020) [31], on the other hand, did not report this complication, nor did Yeo et al. (2016) [18] and López-Vigil et al. (2019) [14].

Postoperative infections were exceptionally rare. Malhotra et al. (2019) [29] identified one case of superficial infection (5%). Some less common complications were also described. McMurrich et al. (2020) [30] reported one case of pulmonary embolism and another of gastrointestinal bleeding, both attributable to the use of nonsteroidal anti-inflammatory drugs and not directly related to the surgical technique. The same study documented the breakage of a scalpel, which was recovered without clinical consequences. Magnan et al. (2018) [28] reported one stress fracture and 11 cases of radiographic misalignment, all of which were asymptomatic. Haque et al. (2016) [25] also described a case of ossification in soft tissues. Salinas-Gilabert et al. (2023) [26] added a case of hammer toe recurrence and ankle discomfort as postoperative adverse events.

In patients with Morton’s neuroma or plantar ulcers, deMeireles et al. (2025) [24] documented a single case of prolonged serous drainage in a diabetic patient, with no other relevant complications. Mehlhorn et al. (2020) [31], in a population with plantar ulcers, identified three cases of ulcer transfer, two recurrences, and one osteomyelitis requiring bone resection, although they reported no surgical infections or structural digital deformities.

Only three cases of plantar hyperkeratosis were recorded, all of which were asymptomatic: two by Krenn et al. (2018) [27] and one by López-Vigil et al. (2019) [14], which showed very favorable results, with no significant clinical complications. Similarly, Wong et al. (2013) [33] and Yeo et al. (2016) [18] reported safe results, with a minimal incidence of complications and adequate functional preservation of the forefoot.

Table 3 shows the complications in quantitative terms.

Prolonged edema was the most common complication, accounting for 30.91% of all reported complications. This high prevalence is consistent with the findings of various studies, such as those by Henry et al. (2011) [16], Biz et al. (2018) [20], Malhotra et al. (2019) [29], Yeo et al. (2016) [18], and Wong et al. (2013) [33]. Secondly, delayed bone healing accounts for 14.90% of complications, appearing repeatedly in studies such as those by Biz et al. (2018) [20], Magnan et al. (2018) [28], Salinas-Gilabert et al. (2023) [26], Neunteufel et al. (2022) [32], and McMurrich et al. (2020) [30].

Transfer metatarsalgia, at 12.73%, ranks third among the most prevalent complications. This biomechanical alteration was described in studies such as those by Biz et al. (2018) [20], Henry et al. (2011) [16], Haque et al. (2016) [25], Malhotra et al. (2019) [29], Magnan et al. (2018) [28], De Prado-Ripoll et al. (2021) [21], Neunteufel et al. (2022) [32], and Salinas-Gilabert et al. (2023) [26]. Floating toe accounts for 10.45% of complications, being predominantly reported in the studies by Neunteufel et al. (2022) [32] and Krenn et al. (2018) [27]. Persistent pain, present in 8.50% of cases, was identified as a relevant complication, although it is often multifactorial and difficult to attribute exclusively to the surgical technique. This complication was particularly mentioned in the studies by Krenn et al. (2018) [27] and Malhotra et al. (2019) [29], among others. Nonunion, although much less frequent, is represented in the graph with 4.55%, in agreement with the studies by Haque et al. (2016) [25], Malhotra et al. (2019) [29], and Krenn et al. (2018) [27]. Finally, the “other complications” category accounts for the remaining 32.27% and includes complications with a low incidence but which, taken together, constitute a high percentage of the total complications. This category includes all other complications: paresthesias, burns, necrosis, stress fractures, infections, embolisms, soft tissue ossification, digital recurrences, major complications, among others, as described in the studies by Biz et al. (2018) [20], Magnan et al. (2018) [28], McMurrich et al. (2020) [30], Mehlhorn et al. (2020) [31], and Salinas-Gilabert et al. (2023) [26].

### 3.2. Assessment of Risk of Bias

The assessment of risk of bias was performed using specific tools developed by the Joanna Briggs Institute (JBI), according to the type of methodological design of each study included. A total of 15 studies were evaluated: 4 prospective cohorts, 10 case series, and 1 single case study. The JBI tools used allow for the analysis of key domains such as participant selection, measurement validity, follow-up, and statistical analysis.

Four studies were evaluated using the JBI Cohort Checklist (see Table 4). Of these, two presented a low risk of bias: Biz et al. (2018) [20] and Magnan et al. (2017) [28], as they complied with most of the items, including valid measurement of exposure and outcomes, sufficient follow-up time, and appropriate statistical analysis. Although Neunteufel et al. [32] did not report clear strategies for managing confounders, its prospective design and consistency in clinical evaluation reduce the overall risk, and it was registered as moderate risk. The study by Mehlhorn et al. (2019) [31] was considered high risk due to deficiencies in the control of confounding factors and follow-up losses that were not fully justified.

The 10 studies classified as case series were evaluated using the JBI Case Series Checklist [35] (see Table 5). Most adequately met the inclusion criteria, clinical reporting, and outcome follow-up. However, two of them, Henry et al. (2011) [16] and Wong et al. (2013) [33], had some major issues: they were not consecutive, did not describe the clinical context well, and had a small sample size. So, these studies were rated as high risk. Others, such as De Prado-Ripoll et al. (2021) [21] and Lopez-Vigil et al. (2019) [14], among others, demonstrated adequate structure and greater comprehensiveness, being classified as moderate to low risk within the design type.

Salinas-Gilabert et al. (2023) [26] and deMeireles et al. (2025) [24] were considered to have a low risk of bias, meeting all criteria.

The only single case study, McMurrich et al. (2020) [30], was evaluated using the JBI Case Report Checklist (see Table 6), meeting all criteria, including a clear description of the patient, intervention, and postoperative outcome. It was therefore considered to have a low risk of bias, although limited by the intrinsic nature of this type of study, which does not allow for generalization or control of variables.

Overall, the results reflect that cohort studies offer greater methodological robustness, while descriptive studies have structural limitations that must be taken into account when interpreting their clinical findings.

In this systematic review, the GRADE system was used to assess the certainty of the evidence provided by the 15 studies investigating the effects of DMMO osteotomy in patients with metatarsalgia. This system considers five key domains: risk of bias, inconsistency between studies, indirect evidence, imprecision of results, and possible publication bias.

Six studies were classified as having moderate certainty, indicating that their conclusions are reliable, although they could change with future research. This group includes studies such as those by De Prado-Ripoll et al. (2021) [21], Neunteufel et al. (2022) [32], and Magnan et al. (2018) [28], which feature rigorous prospective designs, low risk of bias, and the use of validated clinical scales. Well-structured retrospective studies are also considered with moderate certainty, such as those by Biz et al. (2018) [20], Salinas-Gilabert et al. (2023) [26], and deMeireles et al. (2025) [24], which compensate for their limitations with consistent clinical data and the use of tools such as the FFI to assess pain and functionality.

On the other hand, seven studies were rated as low certainty due to more variable methodological quality, the presence of moderate bias, heterogeneity in the results, or poor precision in the presentation of data. This group includes the studies by Malhotra et al. (2018) [29], McMurrich et al. (2020) [30], Krenn et al. (2018) [27], Wong and Kong (2013) [33], Haque et al. (2016) [25], and the comparative studies by Yeo et al. (2016) [18] and López-Vigil et al. (2019) [14]. In the case of Yeo et al. (2016) [18], the lack of randomization and blinding represents a high risk of bias, while in the study by López-Vigil et al. (2019) [14], although clinical and anatomical evaluation are combined, the lack of standardization limits its contribution.

Finally, only one study, that of Yeo et al. (2016) [18], was classified as very low certainty. This study has multiple limitations, including a very small sample (only four patients), the absence of a control group, poor follow-up, and significant methodological weaknesses, which significantly limit the reliability of its conclusions.

Overall, the GRADE analysis indicates that most studies provide acceptable evidence on the DMMO technique. However, the prevalence of observational designs and the lack of randomized clinical trials prevent the certainty from being raised to high levels. Therefore, it is recommended to continue generating studies with more robust and controlled methodologies to consolidate current conclusions and improve evidence-based clinical decision-making (Table 7).

### 3.3. Meta-Analysis Results

**Figure 2:**
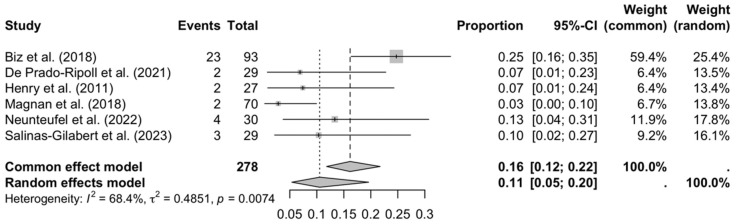
Forest plot: delay in consolidation [16,20,21,26,28,32].

The results of the meta-analysis reveal considerable variability in the incidence of postoperative complications, reflecting methodological, population, and surgical differences between the included studies. In the case of delayed bone healing (see Figure 2), the combined proportion of 11% (95% CI: 0.05–0.20) suggests a moderate incidence, although the significant heterogeneity (I^2^ = 68.4%) could be attributed to factors such as surgical technique, type of osteotomy, or postoperative follow-up. The study by Biz et al. (2018) [20], with a significantly higher proportion (25%), could be influenced by stricter evaluation criteria or specific characteristics of their cohort.

**Figure 3:**
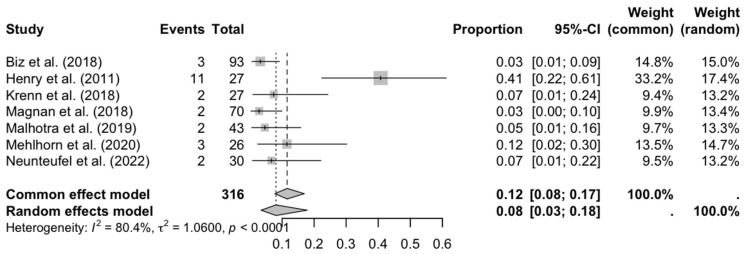
Forest plot: transfer metatarsalgia [16,20,26,27,28,29,31,32].

Regarding transfer metatarsalgia (see Figure 3), the overall proportion was low (8%), but the high heterogeneity (I^2^ = 80.4%) and the identified outlier (Henry et al., 2011 [16]; 27%) raise questions about possible biases or differences in rehabilitation protocols. The asymmetry in the funnel plot reinforces the need for caution when interpreting these results, as studies with lower proportions may be underrepresented.

**Figure 4:**
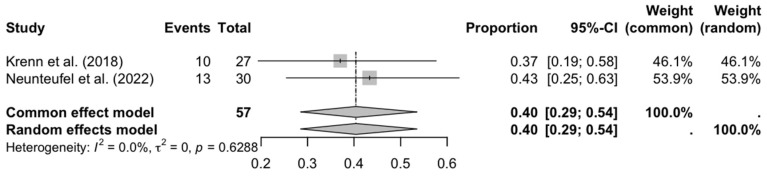
Forest plot: floating toe [27,32].

Floating toe (see Figure 4) showed a high incidence (40%) with consistency between studies (I^2^ = 0%), suggesting that this complication is common and reproducible in different contexts. This could be related to aggressive surgical techniques or inadequate correction of the length of the metatarsal radius.

**Figure 5:**
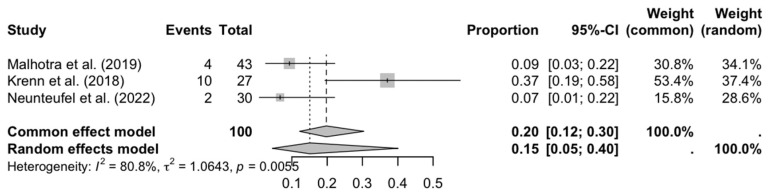
Forest plot: persistent pain [27,29,32].

Regarding persistent pain (see Figure 5), the combined proportion (15%) and significant heterogeneity (I^2^ = 80.5%) indicate that its presentation varies widely, possibly due to differences in the definition of pain, follow-up time, or the effectiveness of analgesic protocols.

**Figure 6:**
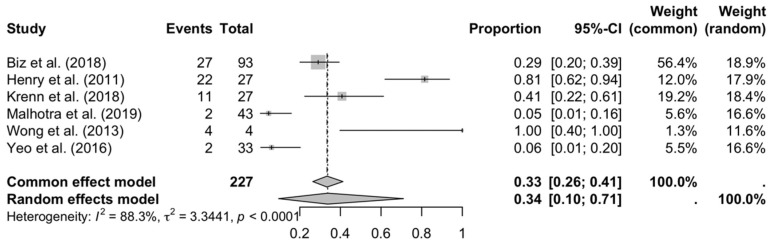
Forest plot: prolonged edema [16,18,20,27,29,33].

Finally, prolonged edema (see Figure 6) showed the greatest inconsistency (I^2^ = 88.3%), with extreme proportions (4–100%), making it difficult to establish an accurate estimate. The inclusion of studies with very short or long follow-ups could explain this variability.

These findings highlight the importance of standardizing diagnostic and methodological criteria in future research, as well as evaluating clinical and technical factors that contribute to complications. The high heterogeneity in most analyses underscores the need for meta-analyses with more studies or the use of regression models to explore sources of variability.

**Figure 7:**
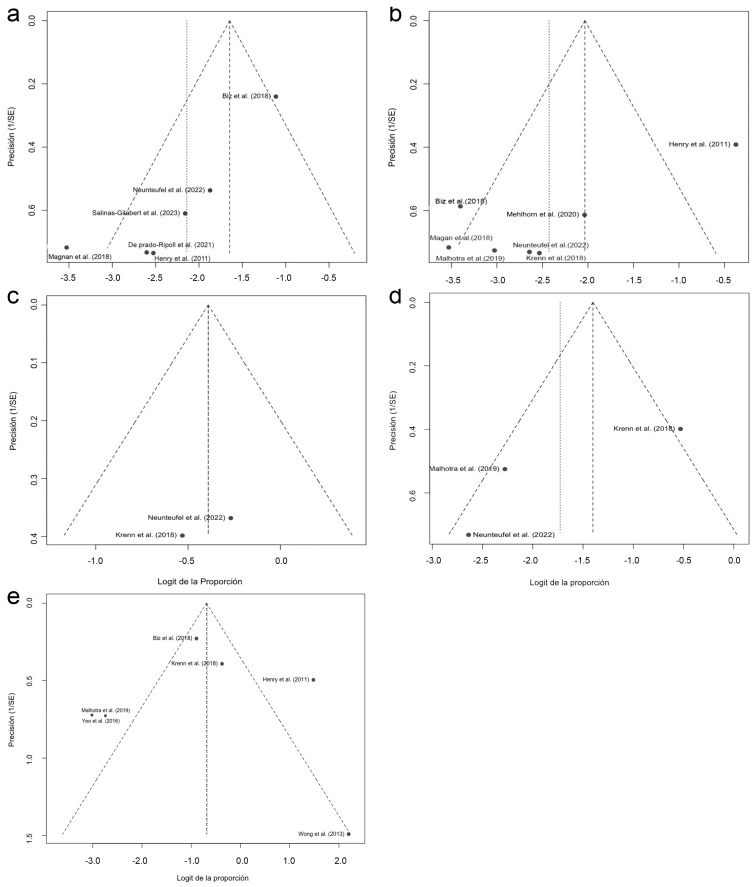
Funnel plots for detecting publication bias in the different analyses of postoperative complications: (**a**) bone consolidation; (**b**) transfer metatarsalgia; (**c**) floating toe; (**d**) persistent pain; (**e**) prolonged edema [16,18,20,21,26,27,28,29,31,32,33].

The assessment of publication bias was carried out by visually inspecting the funnel plots corresponding to each analysis. In general, no marked asymmetries were observed, although interpretation should be made with caution due to the limited number of studies in most meta-analyses. In the case of delayed consolidation (Figure 7a), the distribution was relatively symmetrical, although the study by Biz et al. (2018) [20] shifted slightly to the right, which could reflect a higher proportion of events. In transfer metatarsalgia (Figure 7b), a possible asymmetry influenced by the study by Henry et al. (2011) [16] was evident, suggesting a possible bias or significant clinical variation. For the analysis of floating toe (Figure 7c), the two studies included were distributed symmetrically around the central axis, although the low number of data points limits the usefulness of the graph. Regarding persistent pain (Figure 7d), some dispersion was observed, especially due to the positioning of the study by Krenn et al. (2018) [27], reflecting the statistical heterogeneity of the model. Finally, in prolonged edema (Figure 7e), the funnel plot showed clear asymmetry due mainly to the study by Wong et al. (2013) [33], which reported an extremely high proportion with low precision. Overall, the funnel plots suggest the possible presence of publication bias or methodological differences between studies, especially those with high heterogeneity.

## 4. Discussion

This systematic review compiles the findings of 15 clinical studies that analyzed the DMMO (Distal Minimally Invasive Metatarsal Osteotomy) surgical technique in the treatment of metatarsalgia. Through an evidence-based approach, common patterns in postoperative outcomes were identified, and the most frequent complications were evaluated.

In general terms, the studies show mostly favorable results, especially due to the minimally invasive nature of the technique, which allows for faster functional recovery and less postoperative stiffness, as noted by Yeo et al. (2016) [18]. However, the complication rate varies widely between studies. For example, Biz et al. (2018) [20], in a large study of 93 patients, reported a 24.7% delay in consolidation and 29% postoperative edema, while De Prado-Ripoll et al. (2021) [21] and deMeireles et al. (2025) [24] reported much lower rates, possibly attributed to adequate patient selection and a more focused approach.

Additionally, some of the studies included in this review offer direct comparisons between DMMO and Weil osteotomies, providing valuable insights into complication profiles. Henry et al. (2011) [16] reported a higher incidence of persistent metatarsalgia (29%) in patients treated with the Weil technique compared to those who underwent DMMO. Similarly, Yeo et al. (2016) [18] found that DMMO was associated with better preservation of joint mobility and lower postoperative stiffness relative to Weil osteotomies. These findings suggest that, although both techniques can be effective, DMMO may offer functional advantages and a lower risk of certain complications.

Regarding the complication of floating toe, which was particularly prevalent in the studies by Krenn et al. (2018) [27] and Neunteufel et al. (2022) [32], several technical recommendations have been proposed in the literature to reduce its incidence. These include avoiding excessive shortening of the metatarsal, maintaining appropriate metatarsophalangeal alignment and stability, and ensuring the osteotomy is performed at the correct height. Intraoperative fluoroscopic guidance and careful preoperative planning to balance the forefoot are also emphasized as key strategies to preserve toe function and minimize postoperative digital deformities.

Other studies, such as that by Krenn et al. (2018) [27], reflect a high incidence of serious complications such as pseudoarthrosis (29.6%) and floating finger (37%), associated with the learning curve of the technique. However, more recent studies, such as Ferreira et al. (2022) [17], have reported significantly lower rates of nonunion following the DMMO technique, suggesting that with proper surgical experience and appropriate patient selection, this complication can be minimized. Similarly, Henry et al. (2011) [16] reported prolonged edema in 59% of cases and persistent metatarsalgia in 29%, compared to studies such as that by Lopez-Vigil et al. (2019) [14], where no relevant clinical complications were observed. Magnan et al. (2018) [28] and Malhotra et al. (2019) [29] also documented minor complications, although most were self-limiting or asymptomatic. In contrast, Haque et al. (2016) [25] described more structural complications such as nonunion, soft tissue ossification, and transfer metatarsalgia.

Other studies reflect more controlled risk profiles. Wong and Kong (2013) [33] and Yeo et al. (2016) [18] indicated edema as the only complication, while McMurrich et al. (2020) [30] highlighted isolated systemic events not directly related to the technique. Mehlhorn et al. (2020) [31], in patients with plantar ulcers, observed osteomyelitis without structural deformities, while Neunteufel et al. (2022) did report floating toe in 41.9% of patients, although mostly without clinical reper [32] cussions. Finally, the study by Salinas-Gilabert et al. (2023) [26] combined DMMO with intermetatarsal ligament release, showing mild and resolvable complications.

The results of the meta-analysis show a moderate rate of frequent complications: delayed bone healing (11%) and transfer metatarsalgia (8%), both with significant heterogeneity between studies. The incidence of floating toe was the highest (40%), observed in studies such as those by Krenn et al. (2018) [27] and Neunteufel et al. (2022) [32], while prolonged edema also showed high rates (34%), with studies such as Wong et al. (2013) [33] acting as outliers. Persistent pain was reported in 15% of patients, underscoring the need to improve postoperative and follow-up protocols.

Funnel plots (Figure 2, Figure 4, Figure 6 and Figure 7) show a possible presence of publication bias in some cases, although the limited number of studies reduces the robustness of this assessment. The asymmetry observed, especially in edema and transfer metatarsalgia, could be explained by clinical or methodological variations between studies.

In addition to their use in metatarsalgia, DMMO techniques have also shown excellent results in the treatment of plantar diabetic forefoot ulcers (PDFUs). A separate systematic review found that minimally invasive metatarsal osteotomies yielded a high healing rate of 91.9% and a low recurrence rate of 7.2%, with most complications being manageable, such as moderate edema, asymptomatic nonunions, and localized infections. These procedures reduce plantar pressure, allow early ambulation, and avoid internal fixation, making them especially suitable for patients with compromised tissue healing. Their success in the diabetic population reinforces the functional benefits and safety profile of the DMMO, particularly when performed by experienced surgeons in a multidisciplinary setting.

### Limitations

This review has several limitations that should be considered when interpreting the findings. First and foremost, the absence of randomized controlled trials (RCTs) among the included studies significantly limits the overall strength and level of available evidence. Most of the included studies were observational, primarily case series and retrospective cohorts, which are inherently more susceptible to selection bias, confounding factors, and incomplete outcome data.

Secondly, there was substantial methodological heterogeneity across the studies, particularly in how postoperative complications were defined, measured, and reported. This lack of standardization makes reliable comparisons difficult and weakens the precision of pooled complication rates in the meta-analyses. Furthermore, variability in postoperative follow-up complicates the interpretation of long-term outcomes, as many studies had short or inconsistent follow-up periods that may not reflect late-onset complications.

Potential publication bias was also identified, especially in outcomes with high heterogeneity, as shown in several funnel plots. This bias could result in an overestimation or underestimation of complication rates if studies with negative or unfavorable outcomes remained unpublished.

Another important limitation is the possible influence of the surgical learning curve. Most of the included studies were conducted in specialized centers by surgeons with experience in percutaneous or minimally invasive forefoot techniques. As a result, complication rates may not be generalizable to broader clinical settings or to less experienced surgeons.

A further limitation is the lack of direct comparisons between the DMMO technique and other surgical procedures with the same therapeutic goal, such as open-access osteotomies. Although many of the reported complications, such as delayed union, transfer metatarsalgia, or floating toe, are also observed in other techniques, existing literature suggests that DMMO may be associated with a lower incidence of skin-related and infection-related complications due to its minimally invasive nature. The absence of comparative studies limits the ability to contextualize the performance of DMMO relative to alternative procedures and should be addressed in future research.

Despite these limitations, the findings of this review have relevant clinical implications. By identifying the most frequent and potentially avoidable complications associated with DMMO, this analysis can help guide surgeons in risk assessment, surgical planning, and patient counseling. Additionally, the reported complication rates, while variable, offer a benchmark for clinicians considering DMMO as an alternative to open techniques, particularly when prioritizing faster recovery and reduced soft tissue morbidity. These insights can inform clinical decision-making and highlight the need for better-defined protocols and training to mitigate specific risks such as floating toe or delayed union.

## 5. Conclusions

This systematic review allowed us to identify and analyze the most common complications associated with the DMMO technique. Prolonged edema, delayed consolidation, and transfer metatarsalgia were the most frequently reported complications, followed by floating toe and persistent pain. Most of the included studies were case series, reflecting a limited but consistent level of evidence. The studies reviewed came from different countries, mainly Spain, the United States, and Austria, demonstrating international interest in this technique. In addition, the meta-analysis allowed for more accurate quantification of the incidence of each complication. Overall, DMMO is confirmed as an effective and minimally invasive surgical option, although it requires technical expertise and adequate follow-up to minimize risks. These findings reinforce the need for future research with more robust designs and long-term follow-up.

## Figures and Tables

**Table 1:** PICO table.

Element	Description
P (Population)	Patients diagnosed with metatarsalgia related to structural abnormalities of the forefoot.
I (Intervention)	Minimally invasive surgery, DMMO (Distal Minimally Invasive Metatarsal Osteotomy) technique.
C (Comparison)	No comparison group was included.
O (Outcomes)	Complications of DMMO, surgical failures.

**Table 2:** Description of studies: population, interventions, and outcomes.

Author/Year	Sample (n)	Mean Age (Range)	Follow-Up (Range)	Intervention	Outcomes Assessed	Complications
Biz C et al., 2018 [20]	40 patients (58 feet)	63.4 ± 8.2 years	27.1 ± 7.8 months	Percutaneous distal DMMO of second to fourth metatarsals	AOFAS, pain VAS, satisfaction, radiographs, joint function	Persistent edema (3), superficial infections (2), symptomatic recurrence (2)
De Prado-Ripoll J, De Prado M, Forriol F, 2021 [21]	29 patients	58 years (50–67)	38 months (18–71)	Distal percutaneous metatarsal metaphyseal osteotomies (DMMO)	AOFAS, pain, calluses, joint mobility, alignment, satisfaction	Delayed union (2), recurrence (1), transfer metatarsalgia (1)
deMeireles. et al., 2025 [24]	25 patients	55.8 years (39–76)	17 months (12–27)	Percutaneous distal fourth metatarsal osteotomy + DTML release	VAS (7.35 → 0.41), FFI (57.5 → 17.2)	Prolonged serous drainage (1)
Haque S et al., 2015 [25]	30 patients	60 years (52–72)	>12 months (up to 38)	Minimally invasive DMMO in lesser rays (+/−other surgeries)	MOXFQ, pain VAS, satisfaction	Nonunion (1), transfer metatarsalgia (1), malunion (1), soft ossification (1)
Henry J et al., 2011 [16]	72 patients (DMMO: 39/Weil: 33)	DMMO: 62.3 (35–78)/Weil: 63.2 (43–73)	14.8 months (12–24)	Comparison: percutaneous DMMO vs. open Weil osteotomy	AOFAS, joint mobility, metatarsalgia, satisfaction, X-rays	Delayed union (2 DMMO), transfer metatarsalgia (1 per group)
Lopez-Vigil M et al., 2019 [12]	30 patients	58.9 years (34–87)	1.5 years	Minimally invasive distal osteotomy of second metatarsal	Metatarsal shortening (X-ray), AOFAS, plantar plate integrity	No stiffness, pseudoarthrosis, or delay; asymptomatic hyperkeratosis (3)
Salinas-Gilabert et al., 2023 [26]	29 patients (29 feet)	60 years (43–81)	39 months (25–50)	Percutaneous M2-M3 ligament release + MIS M2–M4 osteotomy	AOFAS-LMTS (50.48 → 88.31), pain VAS, MOXFQ	Delayed union (3), transfer metatarsalgia (1), recurrence (1), hammer toe (1)
Krenn S et al., 2018 [27]	27 patients	60.9 years (48–69)	7.2 months (6.5–8.6)	Minimally invasive DMMO (with/without first ray)	AOFAS, VAS, FAAM, FFI, SF-12, X-rays, pedobarography	Pseudoarthrosis (8), necrosis (4), edema (11), other (several)
Magnan B et al., 2017 [28]	57 patients (70 feet, 106 osteotomies)	60.2 years (30–81)	45 ± 13.3 months (24–68)	Distal percutaneous DMMO (II–IV) without fixation	AOFAS (42.7 → 92.8), satisfaction, alignment, pain, X-ray healing	Delayed union (2), transfer metatarsalgia (2), stress fracture (1), burn (1)
Malhotra K et al., 2018 [29]	28 patients	63 years (43–77)	12 months	Percutaneous DMMO of second to third metatarsals	MOXFQ, satisfaction, global outcome	Delayed union (1), stiffness (1), mild pain (1), infection (1)
McMurrich W et al., 2020 [30]	24 patients	64 years (SD 8.6)	1 year	Distal metaphyseal minimally invasive DMMO (no first ray)	MOXFQ (Walking, Pain, Social), Likert satisfaction	Delayed union (1), GI bleeding + PE (1), burr break (1)
Mehlhorn A.T. et al., 2019 [31]	26 patients	62 ± 9 years	26 ± 18 months (8–43)	Minimally invasive DMMO (MT 2–4 or isolated MT5)	100% ulcer healing rate, 8% recurrence, 11% transfer ulcers	Transfer ulcers (3), recurrence (2), no infection
Neunteufel et al., 2021 [32]	30 patients (31 feet)	63 years	15.5 months (11–25)	Minimally invasive DMMO (rays II–V)	AOFAS, FAOS, FFI, VAS, PPP, shortening, index	Transfer metatarsalgia (2), hyperextension toes (13), delayed union (4)
Wong TC et al., 2013 [33]	4 patients	55.8 years (46–62)	18.5 months (14–24)	Minimally invasive distal metatarsal osteotomy (MIDMO)	Metatarsal index, angles, ACFAS (66.25 → 96.25)	No infection, neurovascular injury, or necrosis. Mild edema (all)
Yeo NEM et al., 2016 [18]	33 patients (Weil: 20/DMMO: 13)	63.8 (Weil)/55 (DMMO)	6 months	Comparison: Weil vs. DMMO for metatarsalgia	AOFAS MTP-IP, pain VAS, RAND-36, ROM	Edema (2), no union delays or failures

**Table 3:** Quantitative table of complications reported in the studies.

Complication	Biz et al. (2018) [20]	Krenn et al. (2018) [27]	Henry et al. (2011) [16]	Neunteufel et al. (2022) [32]	Magnan et al. (2018) [28]	Malhotra et al. (2019) [29]	Salinas-Gilabert et al. (2023) [26]	Mehlhorn et al. (2020) [31]	De Prado-Ripoll et al. [21]	Wong et al. (2013) [33]	Haque et al. (2016) [25]
Delayed healing	23, 0	-	2, 0	4, 0	2, 0	-	3, 0	-	2, 0	-	1, 0
Prolonged edema	27, 0	11, 0	22, 0	-	-	2, 0	-	-	-	4, 0	-
Metatarsalgia transfer	3, 0	2, 0	11, 0	2, 0	2, 0	2, 0	1, 0	3, 0	1, 0	-	1, 0
Floating toe	-	10, 0		13, 0	-	-	-	-	-	-	-
Persistent pain	0, 0	10, 0	1, 0	-	-	4, 0	2, 0	-	-	-	-
Misalignment	-	-	-	-	11, 0	-	-	-	-	-	-
Nonunion	-	8, 0	-	-	-	1, 0	-	-	-	-	1, 0
Recurrence	-		-	1, 0	-	-	1, 0	2, 0	1, 0	-	-
Infection	-		-	-	-	2, 0	-	-	-	-	-
Necrosis	-	4, 0	-	-	-	-	-	-	-	-	-
Hyperkeratosis	-	2, 0	-	-	-	-	-	-	-	-	-
Access port burn	1, 0	-	-	-	1, 0	-	-	-	-	-	-
Dislocation	-		-	-	-	-	-	-	1, 0	-	-
Paresthesia	1, 0	-	-	-	-	-	-	-	-	-	-
Persistent stiffness	1, 0	-	-	-	-	-	-	-	-	-	-
Instrument breakage	-	-	-	-	-	-	-	-	-	-	-
Pulmonary embolism	-	-	-	-	-	-	-	-	-	-	-
Gastrointestinal bleeding	-	-	-	-	-	-	-	-	-	-	-
Bone paste	-	-	-	-	-	-	-	-	-	-	1, 0
Stress fracture	-	-	-	-	1, 0	-	-	-	-	-	-
Surgical resection	-	-	-	-	-	-	-	1, 0	-	-	-

**Table 4:** Assessment of risk of bias in prospective cohort studies according to the JBI Cohort Checklist.

Author and Year	It. 1	It. 2	It. 3	It. 4	It. 5	It. 6	It. 7	It. 8	It. 9	It. 10	It. 11	It. 12
Biz et al. (2018) [20]	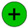	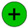	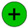	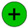	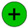	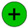	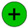	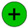	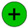	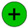	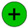	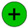
Neunteufel et al. (2021) [32]	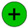	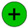	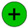	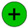	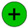	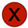	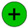	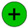	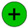	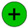	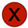	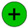
Magnan et al. (2017) [28]	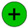	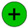	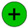	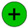	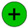	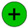	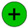	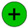	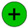	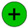	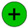	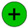
Mehlhorn et al. (2019) [31]	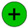	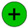	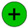	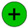	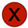	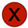	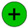	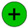	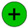	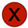	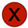	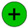

It.1 = Were the two groups similar and recruited from the same population? It.2 = Were the exposures measured similarly to assign people It.3 = to both exposed and unexposed groups? It.4 = Was the exposure measured in a valid and reliable way? It.5 = Were confounding factors identified? It.6 = Were strategies to deal with confounding factors stated? It.7 = Were the groups/participants free of the outcome at the start of the study (or at the moment of exposure)? It.8 = Were the outcomes measured in a valid and reliable way? It.9 = Was the follow up time reported and sufficient to be long enough for outcomes to occur? It.10 = Was follow up complete, and if not, were the reasons to loss to follow up described and explored? It.11 = Were strategies to address incomplete follow up utilized? It.12 = Was appropriate statistical analysis used? “Yes” was represented by the color green and “No” by red.

**Table 5:** Assessment of risk of bias in case series studies according to the JBI Case Series Checklist.

Author and Year	It. 1	It. 2	It. 3	It. 4	It. 5	It. 6	It. 7	It. 8	It. 9	It. 10	Overall Quality
Prado-Ripoll et al. (2021) [21]	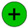	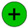	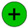	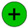	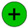	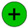	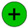	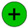	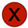	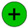	Include
Yeo et al. (2016) [18]	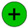	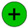	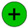	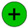	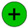	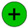	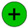	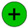	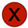	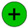	Include
Henry et al. (2011) [16]	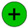	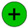	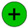	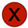	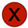	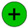	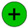	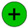	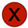	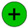	Include
Lopez-Vigil et al. (2019) [12]	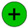	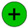	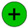	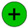	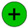	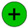	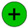	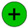	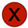	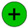	Include
Malhotra et al. (2018) [29]	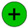	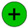	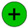	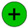	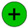	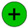	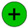	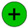	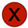	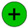	Include
Wong et al. (2013) [33]	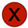	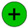	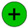	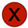	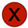	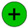	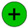	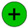	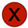	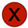	Seek further info
Krenn et al. (2018) [27]	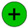	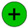	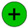	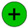	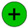	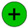	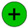	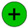	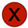	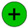	Include
Haque et al. (2015) [25]	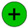	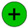	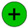	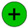	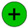	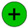	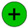	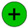	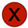	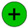	Include
Salinas-Gilabert et al. (2023) [26]	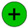	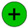	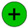	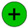	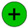	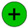	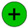	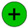	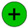	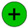	Include
deMeireles et al. (2025) [24]	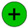	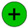	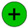	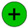	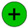	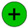	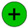	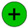	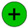	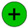	Include

Note: it. 1 = Were there clear criteria for inclusion in the case series?, it. 2 = Was the condition measured in a standard, reliable way for all participants included in the case series?, it. 3 = Were valid methods used for identification of the condition for all participants included in the case series?, it. 4 = Did the case series have consecutive inclusion of participants?, it. 5 = Did the case series have complete inclusion of participants?, it. 6 = Was there clear reporting of the demographics of the participants in the study?, it. 7 = Was there clear reporting of clinical information of the participants?, it. 8 = Were the outcomes or follow-up results of cases clearly reported?, it. 9 = Was there clear reporting of the presenting sites’/clinics’ demographic information?, and it. 10 = Was statistical analysis appropriate?. “Yes” was represented by the color green and “No” by red.

**Table 6:** Assessment of risk of bias in a single case study according to JBI Case Report Checklist.

Author and Year	It. 1	It. 2	It. 3	It. 4	It. 5	It. 6	It. 7	It. 8	Overall Quality
McMurrich et al. (2020) [30]	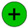	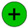	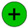	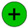	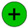	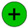	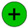	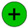	Include

It 1 = Were patient’s demographic characteristics clearly described? It 2 = Was the patient’s history clearly described and presented as a timeline? It 3 = Was the current clinical condition of the patient on presentation clearly described? It 4 = Were diagnostic tests or assessment methods and the results clearly described? It 5 = Was the intervention(s) or treatment procedure(s) clearly described? It 6 = Was the post-intervention clinical condition clearly described? It 7 = Were adverse events (harms) or unanticipated events identified and described? It 8 = Does the case report provide takeaway lessons? “Yes” was represented by the color green.

**Table 7:** Grade.

Author and Year	Design	Risk of Bias	Inconsistency	Indirectness	Imprecision	Publication Bias	Certainty
Biz et al., 2018 [20]	Prospective	Low	Mild	No	No	Not evaluated	Moderate
De Prado-Ripoll et al., 2021 [21]	Prospective	Low	No	No	No	Not evaluated	Moderate
deMeireles et al., 2025 [24]	Case series	High	High	Yes	High	Possible	Very low
Haque et al., 2016 [25]	Retrospective	Moderate	Mild	No	Mild	Not evaluated	Low
Henry et al., 2011 [16]	Comparative retrospective	High	Mild	No	Mild	Not evaluated	Low
Krenn et al., 2018 [27]	Retrospective	Moderate	Mild	No	Mild	Not evaluated	Low
Lopez-Vigil et al., 2019 [12]	Retrospective	Moderate	Mild	Yes	Mild	Not evaluated	Low
Magnan et al., 2018 [28]	Prospective	Low	No	No	No	Not evaluated	Moderate
Malhotra et al., 2018 [29]	Retrospective	Moderate	Mild	No	Mild	Not evaluated	Low
McMurrich et al., 2020 [30]	Prospective	Moderate	Mild	No	Mild	Not evaluated	Low
Mehlhorn et al., 2019 [31]	Prospective	Low	No	No	No	Not evaluated	Moderate
Neunteufel et al., 2021 [32]	Retrospective	Low	Mild	No	No	Not evaluated	Moderate
Salinas-Gilabert et al., 2023 [26]	Retrospective	Low	Mild	No	No	Not evaluated	Moderate
Yeo et al., 2016 [18]	Case series	High	No	No	Mild	Not evaluated	Very low
Wong & Kong, 2013 [33]	Comparative retrospective	High	Mild	No	Mild	Not evaluated	Low

## Data Availability

Data are contained within this article.

## Abbreviations

The following abbreviations are used in this manuscript:

AOFAS	American Orthopaedic Foot and Ankle Society Score
DMMO	Distal Minimally Invasive Metatarsal Osteotomy
DTML	Deep Transverse Metatarsal Ligament
FAAM	Foot and Ankle Ability Measure
FAOS	Foot and Ankle Outcome Score
FFI	Foot Function Index
GRADE	Grading of Recommendations Assessment, Development and Evaluation
HAV	Hallux Abductus Valgus
IP	Interphalangeal
JBI	Joanna Briggs Institute
MIS	Minimally Invasive Surgery
MOXFQ	Manchester–Oxford Foot Questionnaire
MTP	Metatarsophalangeal
PRISMA	Preferred Reporting Items for Systematic Reviews and Meta-Analyses
PROSPERO	International Prospective Register of Systematic Reviews
RX	Radiograph
SF-12	Short Form-12 Health Survey
VAS	Visual Analog Scale

## Appendix A

### Appendix A.1. Search Strings for Electronic Databases

The original search string was created using PubMed. The search strings employed for Web of Science and MEDLINE via PubMed were translated using an automatic online tool https://www.sr-accelerator.com/#/polyglot, accessed on 14 June 2025.

#### Appendix A.1.1. Search Strategy for PubMed

URL: https://pubmed.ncbi.nlm.nih.gov (accessed on 10 June 2025).

((“metatarsal bones/surgery” [MeSH Terms] OR “metatarsalgia/surgery” [MeSH Terms] OR “metatarsalgia” [Title/Abstract]) AND (“minimally invasive surgical procedures/methods” [MeSH Terms] OR “minimally invasive surgical procedures/adverse effects” [MeSH Terms] OR “minimal invasive” [Title/Abstract] OR “percutaneous surgery” [Title/Abstract] OR “surgical procedures” [Title/Abstract]) AND (“osteotomy” [MeSH Terms] OR “osteotomy/methods” [MeSH Terms] OR “osteotomy” [Title/Abstract] OR “osteotomies” [Title/Abstract] OR “elevation osteotomy” [Title/Abstract] OR “metatarsal osteotomy” [Title/Abstract])) AND (“DMMO” [Title/Abstract] OR “distal metatarsal osteotomy” [Title/Abstract] OR (“extracapsular” [All Fields] AND “osteotomy” [Title/Abstract]) OR “intracapsular osteotomy” [Title/Abstract]) AND (“complication*” [Title/Abstract] OR “adverse outcome*” [Title/Abstract] OR “postoperative complication*” [Title/Abstract] OR “surgical failure” [Title/Abstract] OR “preventive strategies” [Title/Abstract] OR “treatment outcome” [MeSH Terms] OR “patient selection” [Title/Abstract] OR “surgical indication*” [Title/Abstract]).

#### Appendix A.1.2. Search Strategy for Web of Science

URL: https://www.webofscience.com/wos/alldb/basic-search (accessed on 10 June 2025).

Filters: No filters were applied.

TS = (“metatarsal bones surgery” OR “metatarsalgia surgery” OR “metatarsalgia”) AND TS = (“minimally invasive surgical procedures methods” OR “minimally invasive surgical procedures adverse effects” OR “minimal invasive” OR “percutaneous surgery” OR “surgical procedures”) AND TS = (“osteotomy” OR “osteotomy methods” OR “osteotomy” OR “osteotomies” OR “elevation osteotomy” OR “metatarsal osteotomy”) AND TS = (“DMMO” OR “distal metatarsal osteotomy” OR (“extracapsular” AND “osteotomy”) OR “intracapsular osteotomy”) AND TS = (“complication*” OR “adverse outcome*” OR “postoperative complication*” OR “surgical failure” OR “preventive strategies” OR “treatment outcome” OR “patient selection” OR “surgical indication*”).

#### Appendix A.1.3. Search Strategy of Scopus

TITLE-ABS-KEY ((“metatarsalgia” OR “metatarsal bones surgery” OR “metatarsalgia surgery”) AND (“minimally invasive surgery” OR “minimal invasive” OR “percutaneous surgery” OR “surgical procedures”) AND (“osteotomy” OR “osteotomies” OR “elevation osteotomy” OR “metatarsal osteotomy” OR “osteotomy methods”) AND (“DMMO” OR “distal metatarsal osteotomy” OR (“extracapsular” AND “osteotomy”) OR “intracapsular osteotomy”) AND (“complication*” OR “adverse outcome*” OR “postoperative complication*” OR “surgical failure” OR “preventive strategies” OR “treatment outcome” OR “patient selection” OR “surgical indication*”)).
